# The effect of electric field intensification at interparticle contacts in microwave sintering

**DOI:** 10.1038/srep32163

**Published:** 2016-09-02

**Authors:** Xiuchen Qiao, Xiaoying Xie

**Affiliations:** 1 School of Resource and Environmental Engineering, East China University of Science and Technology, Shanghai, China

## Abstract

The nature of microwave sintering cannot be explained in the past and has been generally called microwave effect. Here we show that the E-field intensification is the reason of microwave fast sintering of solid state inorganic compounds. The intensification degree varied with dielectric constant of compound, distance between two particles, angle between the direction of E-field and the normal to the surface at the adjacent point of two spheres. Ultra-high temperature caused by E-field intensification leads to fusing of solid materials at contact zone and enhances the mass transportation. The key to develop a microwave energy-saved sintering method is to control the distance between particles and uniformity of particles instead of the particle size.

Microwave irradiation is an innovative technology that dramatically accelerates the reaction rate, shortens the reaction times and energy consumption^1^,^2^,^3^,^4^. With the application of microwave in the preparation of materials, much revolutionary prospects for high-temperature treatment of solid inorganic powder materials have been opened^5^,^6^,^7^,^8^,^9^,^10^,^11^,^12^. Rate-acceleration results which cannot be explained by the dielectric absorption alone, have been regarded as the consequences of so called microwave effect, including the generation of liquid phase at a relatively low observing temperature^13^,^14^, the randomly located “hot spot”^15^,^16^,^17^,^18^ and the fast densification and the “grain growth restriction” in solid powder sintering process^11^,^12^,^19^,^20^. The mechanism of microwave effect in the sintering process of solid powder is still under debating, though different hypotheses have been suggested. Some thought microwave induced an extra driving force to enhance the mass transportation. The driving force caused by microwave selective heating was ascribed to “anisothermal reaction” condition^21^. Agrawal *et al*. believed that “anisothermal effect” was the reason for the observed diffusion enhancement in NiAl_2_O_4_ formation^22^. Through quantitatively theoretical analysis, however, Perry *et al*. proved that the “anisothermal effect” was negligible unless the scale of a particle diameter reached to millimeter scale^23^. Other researchers considered pondermotive force (PMF) as the reason for diffusion enhancement in ionic crystal systems^24^,^25^,^26^,^27^,^28^,^29^. There is positive correlation between PMF and the square of electromagnetic field intensity (PMF∝E^2^). The E-field intensity in a dielectric phase is theoretically ε′ times as low as that in the air in a multi-phase microwave sintering system^30^. It is called electric field (E-field) intensification when the largest E-field intensity in the gap of particles is more than ε′ times as high as that in the dielectric phase. E-field intensification is well known in electrostatics and is suggested to be another reason for microwave effect^16^,^17^,^18^,^31^,^32^. Birnboim *et al*. reported that in ZnO-air system, the E-field intensity in the neck region of two micro-scaled ZnO particles was 30 times as large as the applied field^31^. Booske *et al*. illustrated the E-field intensification at the boundary of two microwave sintered ceramic grains using a geometry and corresponding equations^32^. Though extra driving force or E-field intensification has been generally considered as two different reasons of microwave effect, they should be agreed on the understanding of the same microwave sintering process. As the nature of extra driving force is diffusion enhancement. The diffusion rate is strongly influenced by temperature, which in fact is a reflection of E-field intensity under microwave irradiation. However, the E-field intensification had not been proven as a universal phenomenon in dielectric-air and metal-air systems. Questions such as “when it would occur” and “how to control it” are still unknown, which hinder the development of microwave sintering of inorganic materials.

In this report, micrometer and millimeter scaled E-field intensification of microwave was investigated in dielectric-air and metal-air systems. The influence of dielectric constant (ε′) and distance between different scaled specimens on the E-Field intensification were presented by means of simulation and experimental methods. A conclusive explanation on microwave effects was obtained and a more scientific utilization of microwave irradiation in sintering process would be expected.

## Results

### E-field distribution around two particles

Researchers have already found that E-field intensification in two micrometer scaled ceramic spheres is determined by the angle between the direction of E-field and the normal to the surface at that contact point of two spheres^31^. The intensification disappears when the angle increases from 0° to 90°. Besides angle, dielectric constant ε′ and the distance between particles would play crucial parts in E-field intensification. The ε′ of a material shows its polarizability caused by external E-field and determines its absorption ability of microwave.

**Figure 1:**
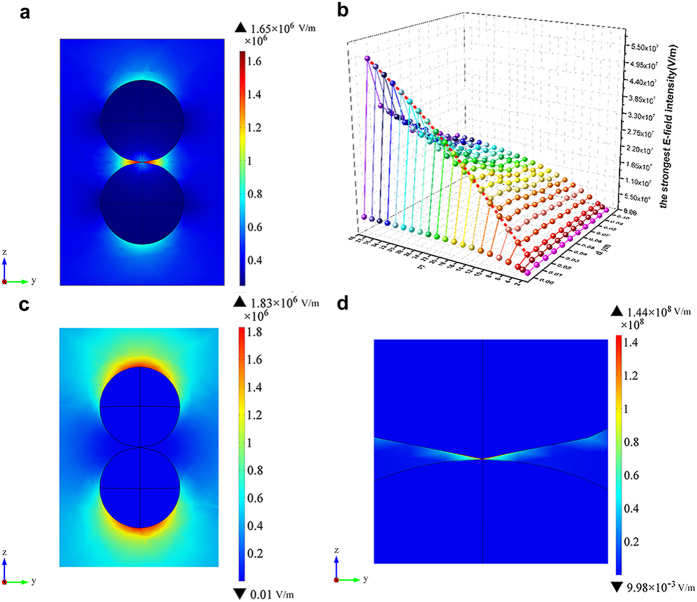
E-field intensification occurred in two dielectric spheres. (**a**) Distribution of E-field intensity around two contact dielectric spheres. (**b**) Strongest E-field intensity in the gap between dielectric spheres with a ε′ varied from 2 to 40, the distance between spheres ranged from 0 to 0.1 μm. (**c**) Distribution of E-field intensity around two contact copper spheres. (**d**) Distribution of E-field intensity around two copper spheres with a distance of 0.01 μm.

Simulation results of the two 10 μm-dielectric sphere model showed that the E-field energy was strongly “collected” around the contact point of two spheres due to E-field intensification (Fig. 1a). The intensification degree of E-field varied with the distance between two dielectric spheres and the largest E-field intensity presented in the middle of two spheres (Fig. 1b). The strongest intensification always occurred at the distance of 0.01 μm even with variation in ε′. When ε′ was larger than 4, the “collected” E-field was strong enough (>3 × 10^6^ V/m) to ionize air in the gap of spheres. The largest E-field intensity in the gap increased with ε′, however, the increase ratio of E-field intensity slowed down.

When the dielectric spheres were changed to two identical cooper spheres, the E-field did not intensify at the contact zone of two metal spheres (Fig. 1c). However, it strongly intensified with the departure of two copper spheres and the strongest intensification occurred at the same distance of 0.01 μm (Fig. 1d). The largest E-field intensity was up to 1.4 × 10^8^ V/m. Although the E-field intensified in the gap of two metal spheres, the E-field intensity inside the metal spheres kept unchanged.

Simulation results illustrate the fact that E-field intensification occurs in the gap of two dielectric and metal particles, which would contribute to the anisothermal heating of materials and the appearance of “hot spots”. The intensified E-field energy of microwave in the gap of metal particles is one to two orders of magnitude higher than that of dielectric particles, which is able to ionize air and induces microwave plasma sintering of metal particles. However, the E-field intensification does not occur when metal particles connect to each other. It may be a practical solution to heating metal powder compacts through microwave irradiation that covering metal particles with a thin insulating layer such as native oxide^33^.

### E-field distribution in compacted powders

**Figure 2:**
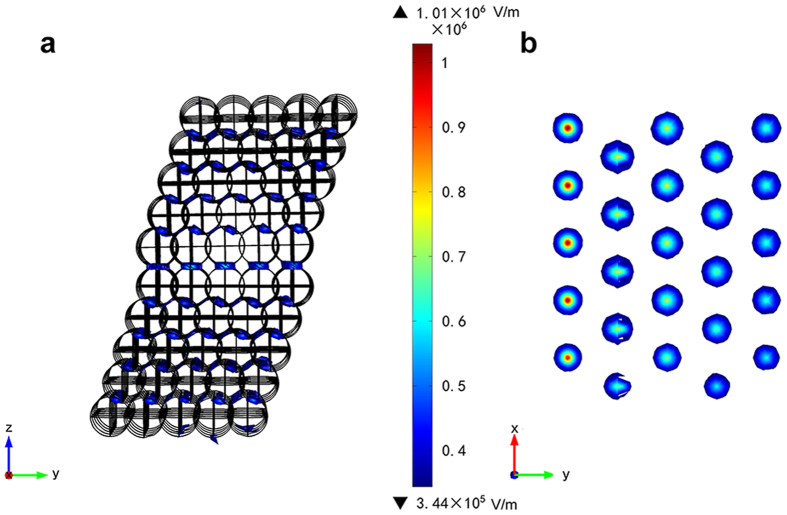
Distribution of E-field intensity in compacted particle model. (**a**) E-field intensity varied from 3.44 × 10^5^ V/m to the maximum in compacted model. (**b**) Distribution of E-field intensity of the plane that crossed the junction of two arrays along axes x and y.

In most conditions, powder materials are compacted before sintering. For simplicity, two 5 × 5 × 5 cubic closest-packed 10 μm-sphere arrays were built. As coal fly ash (CFA) is an originally spherical particle and is easier to prepare larger scaled specimens in the subsequent experiment. Here the ε′ of CFA, i.e. 3.34, was applied to dielectric spheres in arrays. An angle of 1° between two arrays was designed to investigate the influence of distance on the E-field intensification (Extended Data Fig. 1). Simulation results showed that the average E-field intensity inside the CFA spheres was 1.03 × 10^5^ V/m. The intensity illustrated in Fig. 2a was set from 3.44 × 10^5^ V/m (3.34 times as much as 1.03 × 10^5^ V/m) to 1.01 × 10^6^ V/m (maximum) in order to show only the phenomena of E-field intensification. The E-field energy intensified in many spots with different intensity around contacted pointes of CFA spheres. When the angle between the direction of E-field (parallel to axis z) and the normal to the surface at the joint of two arrays was 0°, the strongest E-field intensification occurred. At the contact point of each CFA sphere inside the two arrays, the angle was 35.3°, the E-field intensification degree was relatively weak. When the angle was 90°, the intensity of E-field between two spheres was lower than 3.44 × 10^5^ V/m and the adjacent regions were colored white in Fig. 2a. On the plane that crossed the junction of two arrays along axes x and y, the E-field intensity around two CFA spheres decreased with the increase in distance of two arrays along axis z (Fig. 2b).

**Figure 3:**
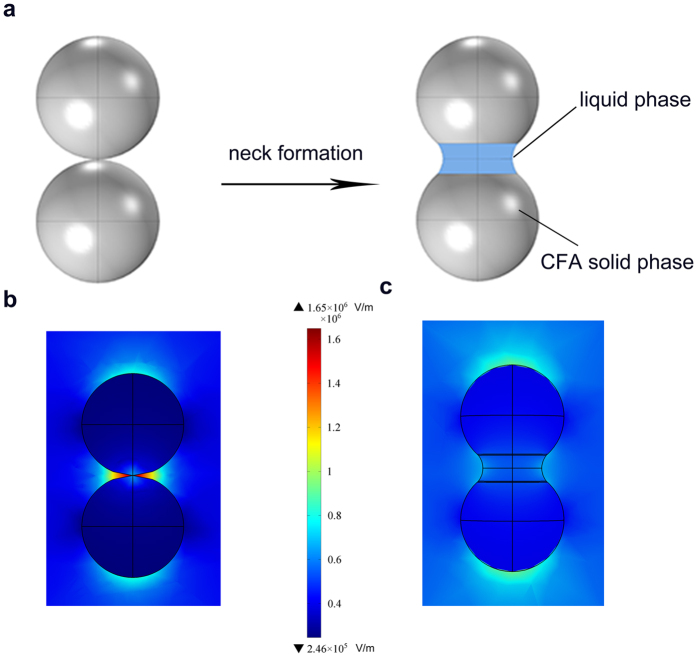
Schematic diagram of neck growth and distribution of E-field intensity at the neck zone. (**a**) Growth of neck zone. (**b**) Intensification of E-field before the formation of neck zone. (**c**) Disappearance of E-field intensification after the formation of neck zone.

Results in Figs 1 and 2 showed that E-field intensification occurred in the compacted dielectric-air models and the distribution of E-field intensity was non-uniform. High temperature caused by strong E-field intensification would, therefore, lead to fast formation of liquid phase and neck growth between particles (Fig. 3a). With the extrusion of air between dielectric particles by liquid phase, E-field intensification disappears (Fig. 3b,c). Rybakov *et al*. and Olevsky *et al*. provided an insight into the neck growth driven by PMF which enriches the understanding of the performance of E-field intensification^28^,^29^. Their results showed that the additional compressive pressure due to PMF would decrease with the decrease of E-field intensification and the increase of temperature. Therefore, microwave effects are mostly observed at the initial stage of sintering.

The dielectric materials are poor conductor of heat. And the polarization ability of dielectric materials increases with the transformation from solid to liquid. The fused dielectric materials can continuously absorb microwave to enhance sintering even after the disappearance of E-field intensification. This is the reason for fast and anisothermal sintering of dielectric powders. However, the metal materials are good conductor of heat, which leads to instant cool down in temperature of melted metal at neck zone after the disappearance of E-field intensification. Dramatically high temperature caused by microwave plasma contributes to the fast formation of low viscosity liquid phase of metal. After the decrease in temperature, more grain cores and small size crystals correspondingly form, i.e. restriction of grain growth.

Microwave-assisted organic synthesis, which is designed to achieve high systematic dielectric loss by the utilization of polar solvent, is thought to be a green chemistry and applied worldwide in the synthesis of materials. Solid state inorganic materials, however, are generally of low dielectric loss or difficult to be polarized, which twists the application of microwave in the sintering of inorganic materials. Therefore, the hybrid heating, for instance introduction of SiC, was developed. However, it is unwise to sinter powder materials by hybrid heating under microwave irradiation, which stops the most crucial effect of microwave, i.e. E-field intensification.

### E-field distribution around cylinder specimens

**Figure 4:**
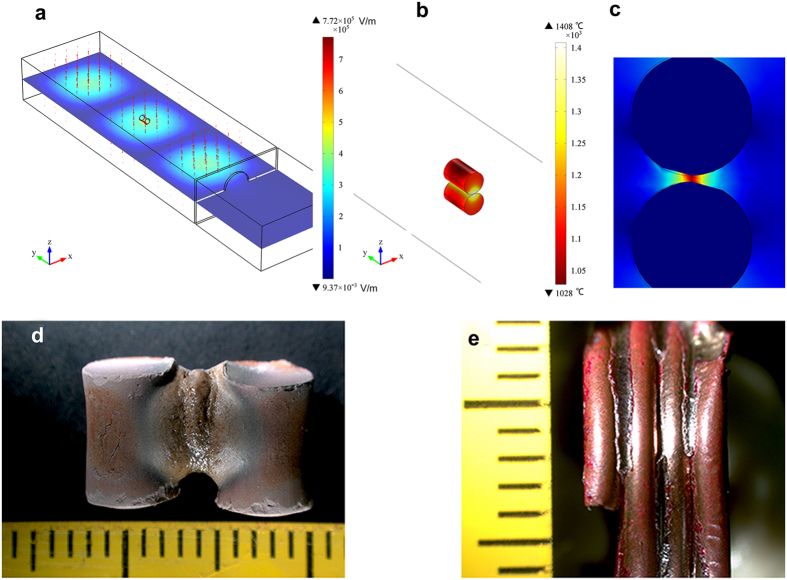
E-field intensification occurred in the gap of cylinder samples. (**a**) Distribution of E-field intensity around two CFA cylinder samples. (**b**) Temperature distribution of CFA specimens irradiated for 6 s. (**c**) Distribution of E-field intensity around two copper cylinder samples. (**d**) CFA cylinder samples sintered at an input microwave power of 250 w for 100 s. (**e**) Copper cylinder specimens sintered at an input microwave power of 500 w for 60 s.

To clearly observe the effect of E-field intensification in micrometer scaled specimen, the sintering of two dielectric cylinders (5.5 × 8 mm) made of CFA and two copper cylinders (1.4 × 10 mm) were also simulated in the TE_103_ cavity, respectively. E-field intensification occurred when the direction of E-field was parallel to the normal to the surface at the joint of two cylinders. The largest E-field intensity of 7.84 × 10^5^ V/m appeared at the contact boundary of two CFA specimens under the input microwave power of 250 W (Fig. 4a). The calculated temperature at the contact boundary reached over 1400 °C in a few seconds (Fig. 4b), which was high enough to melt CFA. The largest E-field intensity of 3.06 × 10^6^ V/m presented under the irradiation of 500 w microwave when the distance between two copper cylinders was 80 μm (Fig. 4c).The intensified E-field was large enough to ionize air to melt copper. Based on the simulation results the same size of CFA cylinder and copper cylinder specimens were prepared and irradiated in the TE_103_ single mode microwave applicator equipped with IR thermal imaging system that detects only the surface temperature of specimen^13^,^34^. When the specimens satisfied the E-field intensification requirements, large amount of fused phase formed in the gap of two CFA cylinder specimens (Fig. 4d). The conditions of generating liquid phase in the experiment, therefore, correspond well with simulated results. However, the highest surface temperature at the joint of these two CFA specimens was about 700 °C. The fused phase also formed between copper cylinder specimens (Fig. 4e) and the IR camera did not detect any signal of temperature higher than 200 °C. The significant heat loss on surface is one reason for the difference between monitoring temperature and melting temperature, however, it may not be the only factor. As the increased compressive stress due to PMF in the gap of cylinder specimens may be another reason^29^. This phenomenon can be a subject for future investigation.

When the direction of E-field was perpendicular to the normal to the surface at the joint of two cylinders, the phenomenon of E-field intensification disappeared.

## Discussion

The key of microwave sintering in the past have been considered as the control of particle size, especially after the extraordinary investigation of Roy *et al*.^9^ on full microwave sintering of micro-scaled powdered metal. Therefore, the investigation of microwave sintering has been limited on micro-scaled materials, which made it hard to directly observe the E-field intensification. Here we show that the truth of microwave sintering of inorganic solids is E-field intensification, which would occur at various scales in both dielectric-air and metal-air sintering systems. The key of microwave sintering is to control the distance between particles and the angle between the direction of E-field and the normal to the surface at the joint of two particles. The mass transportation between two particles will be enhanced along the direction of E-field due to an instant high temperature caused by E-field intensification.

The nature of microwave effect in the sintering of inorganic powder materials, i.e. E-field intensification, is concluded from the simulations and experiments operated in a single mode cavity. The E-field intensification will occur more frequently when the distances between particles are appropriate in a multi-mode cavity of microwave, because the probability of the normal to the surface at every two adjacent particles paralleling to the direction of E-field is higher. The harmful “hot spot” in inorganic synthesis can be avoided through the control of uniformity of particles. This investigation will help scientists and engineers to develop the utilization technologies of microwave irradiation in sintering process of inorganics.

## Methods

### Preparation of cylinder samples

A Class F coal fly ash (CFA) was collected from Shanghai Wujing coal fired power plant. This CFA had an average particle size of 10 micrometers analyzed by Malvern analyzer and mainly consisted of spherical particles. A batch of two samples of 40 g of dry CFA and 20 ml of ethanol were pulverized for 2 h in a planetary ball mill equipped with two 500-ml agate jars. The wet milled sample was oven dried at 105 °C for 24 h, then pressed to form cylindrical specimens of 5.5 × 8 mm. The cylinder copper specimen was prepared by bending a 1.4 mm solid round copper wire into a helix with a pitch of 0.08 mm and the ends were carefully welded together to avoid the influence of sharp ends.

### Experimental apparatus

The illustration of experimental apparatus was shown in previous investigation^34^. The waveguide (Extended Data Fig. 1) cavity TE_103_ was connected to a power source through a coupled aperture with a diameter of 25 mm.

### Microwave irradiation of specimens

All the specimens were located in the center of a microwave transparent insulation carrier^34^, and then mounted at the center of cavity, where the H-field effect was negligible. During microwave irradiation, the sliding short circuit piston was first adjusted to the position with minimal output power for the best coupling and then held still for required time. The input and output power were measured using power monitors. The difference between the input and output powers was the absorbed power for heating samples. CFA cylinders were irradiated under an input power of 250 w for 100 s. Copper helix was irradiated under an input power of 500 w for 60 s.

### Simulation of E-field intensification

Microwave irradiation was simulated using RF module of COMSOL Multiphysics software (Ver 4.2a). The copper waveguide operates at a frequency of 2.45 GHz. The skin depth of copper is much smaller than the dimensions of the waveguide. The electromagnetic losses can be localized entirely on the surface, i.e. the power loss in the waveguide wall can be ignored. Thus, Maxwell’s equations were solved only in the waveguide. The rectangular port was excited by the fundamental TE_10_ mode. At the port, the boundaries were transparent to the TE_10_ mode. The relative permeability of all samples are 1. The conductivity of copper is 5.998 × 10^7^ (*S*/*m*). The relative dielectric permittivity of compacted CFA cylinder is 2.30, analyzed by a cylindrical cavity resonating at 2.45 GHz and TEM_010_ (transverse electric wave) mode. The relative dielectric permittivity of CFA sphere particle is 3.34 after eliminating the effect of the porosity using equation 1^35^.


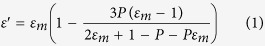


where *ε′* is the relative dielectric permittivity of the cylinder sample that is a mixture of CFA particles and air. *ε*_*m*_is the relative dielectric permittivity of CFA particle, *P* represents the porosity of cylindrical sample, which is determined by the weight and density (*g*/*cm*^*3*^) of the powder materials.

The distribution of E-fields is described through Maxwell’s equations. To satisfy the experimental conditions, the length of waveguide was adjusted to coupling condition through parameter scanning while simulating the E-field intensification of cylinder specimens. The cylinder specimens consisted of 760 tetrahedrons with the smallest mesh size of 10 μm.

A two-sphere model was designed to simulate the E-field distribution of dielectric material-air system. Two spheres of 10 μm diameter were placed next to each other and mounted at the center of TE_103_ cavity with a 200 w input power. The direction of E-field was parallel to the normal to the surface at the joint point of two spheres. The *ε′* of dielectric sphere was initialized from 2 to 40 with a step of 2. The surface meshes of two spheres consisted of approximate 1200 tetrahedrons. To be more accurate, a virtual rectangular cuboid with the smallest mesh size of 5 nm was designed to cover two spheres. The mesh size outside the cuboid was 5 times as small as the wavelength, i.e. 0.024 m.

To simulate the microwave sintering process of compacted powder materials, two 5 × 5 × 5 cubic closest-packed 10 μm-sphere arrays were built. An angle of 1° between two arrays was designed to investigate the influence of distance on the E-field intensification (Extended Data Fig. 2).

### Heat transferred from microwave

The heat transferred from microwave was calculated using following equation.


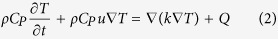


where *ρ* is the density of samples, *C*_*p*_ is heat capacity at constant pressure, u is the velocity field, *k* is the thermal conductivity of the specimens, *Q* represents the electromagnetic losses, which in our systems only includes the resistive losses *Q*_*rh*_.


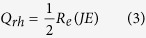



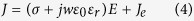


where J is current density, σ is electrical conductivity, ω is angular frequency of wave, ε_0_ is the permittivity of vacuum, ε_r_ is the relative permittivity of materials, Je represents the externally generated current, which is zero in our case. The variations of *C*_*p*_ and *k* with temperature are shown in Extended Data Fig. 3. The density of CFA cylinder is 1750 *kg*/*m*^3^, the density of CFA particle is 2600 *kg*/*m*^3^, the imaginary part of dielectric constant of CFA is 0.1.

## Additional Information

**How to cite this article**: Qiao, X. and Xie, X. The effect of electric field intensification at interparticle contacts in microwave sintering. *Sci. Rep.* **6**, 32163; doi: 10.1038/srep32163 (2016).

## Supplementary Material

Supplementary Information
