# Supplementary material for: The effect of electric field intensification at interparticle contacts in microwave sintering
Source: Sci Rep. 2016 Sep 2;6:32163. doi: 10.1038/srep32163 (PMC5009377; doi:10.1038/srep32163)
Supplement: Supplementary Information [file srep32163-s1.pdf]

The effect of electric field intensification at interparticle contacts in microwave sintering

Xiaoying Xie, Xiuchen Qiao*

Supplementary information

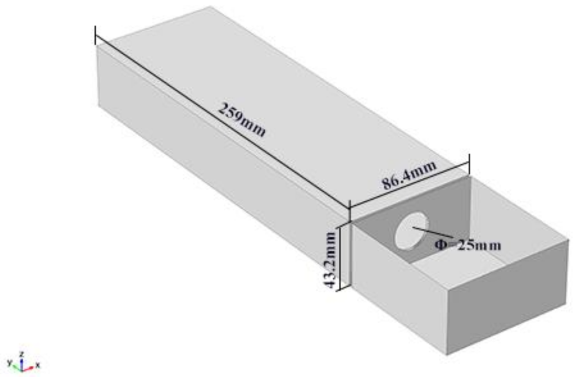

Supplementary Figure S1. Schematic Illustration of TE103 waveguide

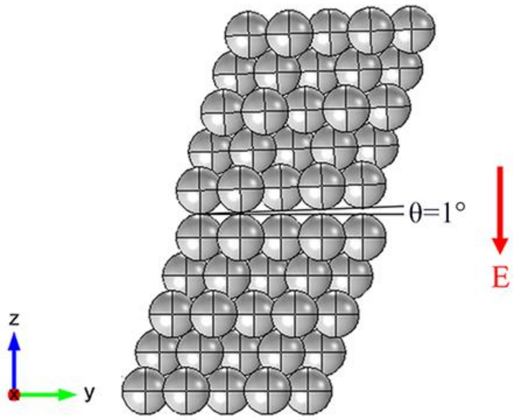

Supplementary Figure S2. Two cubic closest-packed CFA arrays with an angle of 1 °.

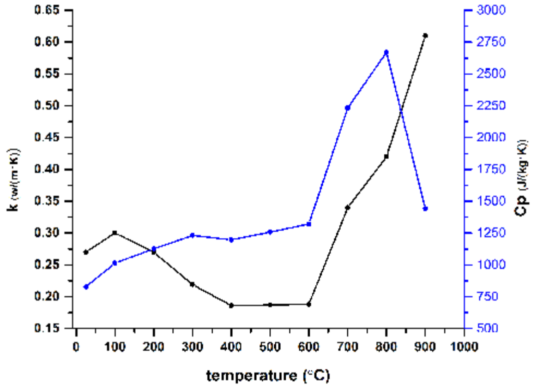

Supplementary Figure S3. Variations of thermal conductivity (*k*) and heat capacity (C_p) of CFA cylinder specimen with temperature at atmospheric pressure.
